# Research hotspots and emerging trends of pain neuroscience education in chronic pain management: A bibliometric visualization study

**DOI:** 10.1097/MD.0000000000050742

**Published:** 2026-09-25

**Authors:** Yixin Chen, Huamei Yu, Lixian Dang, Lingling Li, Chenyang Li

**Affiliations:** a School of Nursing, Inner Mongolia Medical University, No. 5 Xinhua Street, Hohhot, Inner Mongolia Autonomous Region, China; b Department of Nursing, The Affiliated Hospital of Inner Mongolia Medical University, No. 1 Tongdao North Street, Huimin District, Hohhot, Inner Mongolia Autonomous Region, China.

**Keywords:** chronic pain, CiteSpace, pain neuroscience education, research hotspots, visualization analysis

## Abstract

**Objective:**

Chronic pain is a major global public health concern. Pain neuroscience education (PNE), a cognitive intervention grounded in the biopsychosocial model, has been increasingly applied to chronic pain management. However, its knowledge structure and research evolution have not been systematically delineated. This bibliometric study aimed to map the research landscape, identify hot topics and emerging trends, and provide evidence for clinical translation and research planning.

**Methods:**

Publications on PNE in chronic pain were retrieved from the Web of Science Core Collection (2015–2026). A total of 409 articles were analyzed using Microsoft Excel, CiteSpace, VOSviewer, Bibliometrix, and SCImago Graphica to examine publication outputs, collaboration networks, keyword co-occurrence, clustering, burst detection, and citation patterns.

**Results:**

Annual publications increased rapidly from 2018 to 2024, peaking in 2024. The United States, Spain, and Belgium were the most productive countries, with strong collaboration between North America and Europe, while Asian and low-and middle-income countries remained peripheral. Jo Nijs team was a central research force. Keyword analysis revealed 3 hotspots: predominant focus on chronic musculoskeletal pain; a shift from single PNE to multimodal regimens combining exercise and cognitive behavioral therapy; methodological validation of assessment tools serving as a critical hub linking research branches. Burst analysis indicated a transition from empirical accumulation to evidence synthesis with personalized interventions for older adults and adolescents as future frontiers.

**Conclusion:**

PNE research is advancing rapidly, shifting from efficacy validation toward implementation science and precision interventions. Current research shows a geographic imbalance; future efforts should enhance cross-regional knowledge translation. This study provides a clear knowledge map for researchers and evidence-based directions for optimizing pain management systems.

## 1. Introduction

Pain is an individual experience influenced to varying degrees by biological, psychological, and social factors,^[1]^ and chronic pain is defined as persistent or recurrent pain lasting longer than 3 months.^[2]^ It is a major public health issue worldwide, leading to disability, reduced quality of life, and an increased healthcare burden.^[3]^ The traditional biomedical model is insufficient to address chronic pain, prompting a shift in clinical practice and scientific research toward a more holistic biopsychosocial model.^[4,5]^ Although pain education has been incorporated into various chronic pain management guidelines, current approaches are largely based on the biomedical model and focus on explanations of tissue damage.^[6]^ This approach may lead patients to focus excessively on anatomical causes, thereby inducing or exacerbating pain-related anxiety and kinesiophobia. Consequently, a new educational approach known as pain neuroscience education (PNE) has emerged. Rather than a 1-way transfer of knowledge, PNE is a cognitive restructuring intervention grounded in the theory of neuroplasticity. It aims to teach patients modern neurobiological principles of pain, such as central nervous system sensitization and neuroplasticity, to help them reconstruct their scientific understanding of pain, correct erroneous beliefs at their roots, and improve pain intensity, functional impairment, and quality of life.^[7–9]^ Compared with traditional pain education, PNE offers the advantages of a more robust theoretical foundation, more precise intervention targets, and more stable long-term efficacy. PNE has become a cornerstone strategy in modern pain management by reducing the perceived threat of pain and promoting adaptive coping,^[10]^ and has been proven effective within the biopsychosocial framework, with its application now extending into public health domains.^[11,12]^ Despite growing scholarly attention, existing syntheses of PNE research rely primarily on narrative reviews or condition-specific meta-analyses,^[13,14]^ which, while clinically informative, lack the capacity to map the intellectual structure of the field, trace knowledge diffusion across disciplines, or discern longitudinal conceptual evolution. Bibliometric methods can effectively address these limitations by quantitatively analyzing publication trends, co-occurrence networks, and emerging patterns.^[15,16]^ However, existing bibliometric studies on chronic pain management have mostly focused on specific therapies^[17]^ or particular types of pain,^[18]^ lacking systematic knowledge mapping of PNE as a distinct educational intervention model. To address this gap, this study utilized Microsoft Excel, CiteSpace, VOSviewer, Bibliometrix, and SCImago Graphica software to conduct a visual analysis of relevant literature on the application of PNE in the management of patients with chronic pain from the Web of Science Core Collection (WoSCC). It examined characteristics such as publication trends, international collaboration networks, and keyword co-occurrence relationships to identify research hotspots and frontiers in the field, thereby providing guidance for selecting future research directions and offering insights for optimizing clinical pain management practices.

## 2. Materials and methods

### 2.1. Data sources and search strategy

This study used the WoSCC as the data source and employed subject-based search. Based on the usage of specialized terminology in the field, the following subject search strategy was formulated: topic search = (chronic pain OR widespread chronic pain OR chronic primary pain OR low back pain OR neck pain OR fibromyalgia OR osteoarthritis pain OR headache) AND topic search = (pain neuroscience education OR pain biology education OR therapeutic neuroscience education OR neurophysiology education OR pain neurophysiology education). A landmark relevant study^[19]^ published in 2014 expanded readers’ the understanding of pain mechanisms and facilitated the emergence of modern educational interventions. Since then, global research on PNE has undergone rapid and standardized development. Recent literature was selected to reflect the latest research developments and trends in the field, and the time scope of included publications was set from January 1, 2015, to June 1, 2026; the formal literature retrieval was performed on June 28, 2026. Language was restricted to English, and document types were limited to original research articles and review papers.

### 2.2. Inclusion and exclusion criteria

Inclusion criteria: studies that focused on the application of PNE for chronic pain; studies involving participants who met the definition of chronic pain (pain lasting ≥ 3 months); literature from which complete bibliographic information could be extracted. Exclusion criteria: duplicate publications; nonacademic reports such as conference papers and newspaper articles, as well as reports clearly inconsistent with the scope of this study; non-English language literature; studies that only discussed the basic theories of PNE without addressing clinical applications in chronic pain; studies with incomplete information or from which valid data could not be extracted.

### 2.3. Literature screening

This study integrated Microsoft Excel, CiteSpace 6.4.R1, VOSviewer 1.6.21, the Bibliometrix R package (version 4.6.1), and SCImago Graphica (version 1.0.55; SCImago Lab) for comprehensive quantitative statistics and visual mapping. Bibliographic information was exported in plain text format, and 2 researchers independently conducted literature retrieval and screening. In cases of disagreement, discussions were held to reach consensus. Inter-rater reliability was assessed using the kappa statistic, with a kappa value of 0.84 (*P* < .001). The retrieved literature was imported into CiteSpace and VOSviewer, and the deduplication function was used to remove duplicate entries. A flowchart of the literature collection and data analysis process is shown in Figure 1.

**Figure 1.**
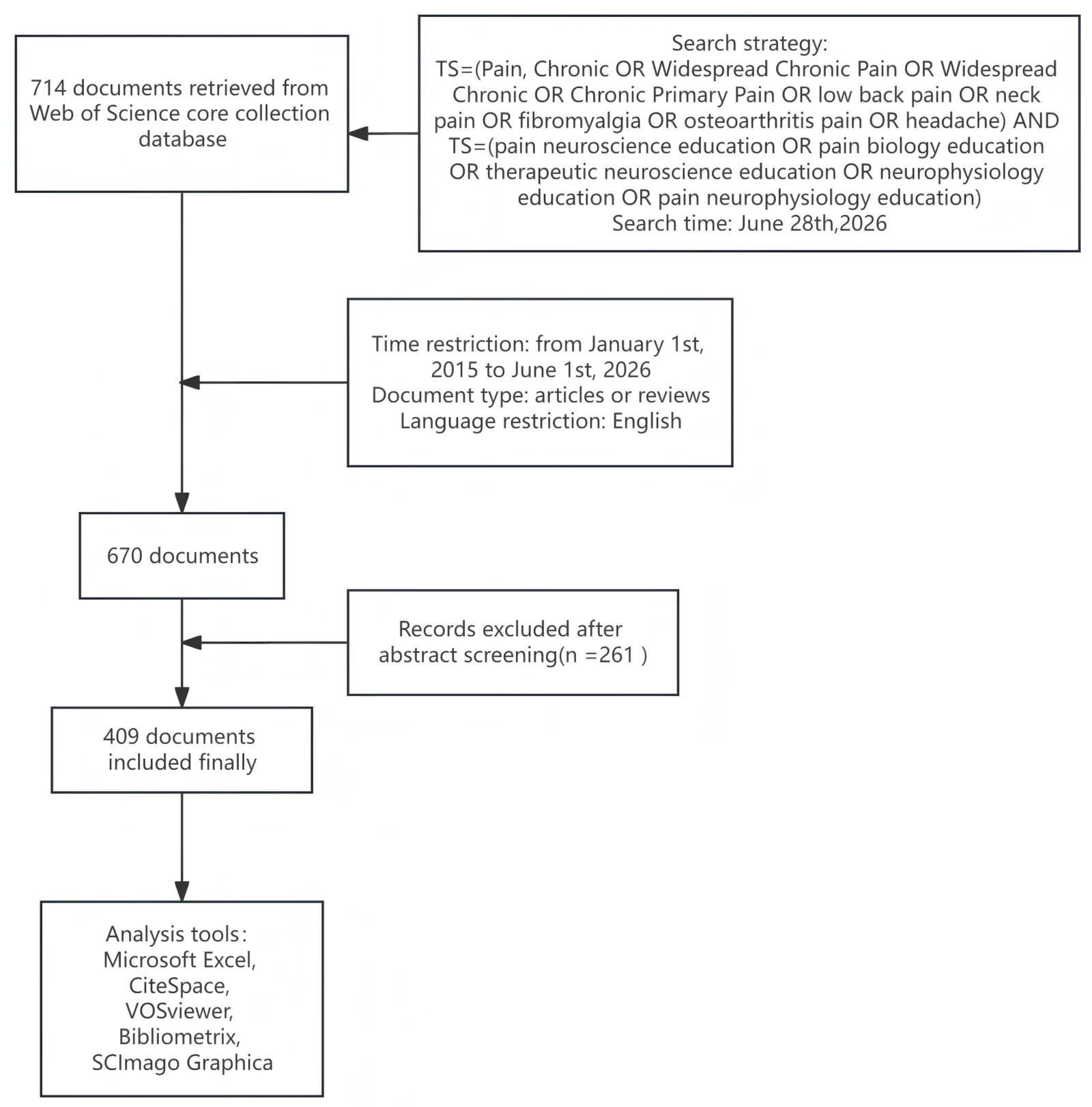
Literature screening flowchart.

### 2.4. Parameter settings

Separate parameter configurations were customized for each analytical tool.

Within CiteSpace, the Web of Science time slice was set to 1 year, and nodes were set to authors, keywords, and cited references. Sensitivity tests with a g-index of *k* = 20, 25, and 30 were conducted to avoid threshold bias, and *k* = 25 was selected for stable network visualization. The pathfinder and 2 pruning strategies were applied to the keyword networks, and all other parameters were kept at their default values. Node size, color, and links represented frequency, publication year, and relational connections, respectively.

In VOSviewer, the minimum occurrence thresholds were set to 3 for countries, 5 for authors, and 5 for keywords to remove low-impact nodes.

Standardized bibliometric text files were uploaded to Biblioshiny (Bibliometrix 4.6.1) for quantitative statistics and auxiliary plotting. Microsoft Excel generated the annual publication volume chart, whereas SCImago Graphica was employed to draw international and author collaboration networks under default built-in parameters.

## 3. Results

### 3.1. Publication trends

A total of 409 valid publications were ultimately included in this study. After the statistical analysis of the included publications by year was conducted, a publication volume chart was generated using Excel, as shown in Figure 2. The annual publication volume data indicate that the field has shown an overall upward trend since 2015, with a marked acceleration after 2018, reaching a peak in 2024. The literature search for this study was conducted up to June 28, 2026, and only publications from the first half of 2026 were counted, resulting in a temporary decline in annual publication output for that year.

**Figure 2.**
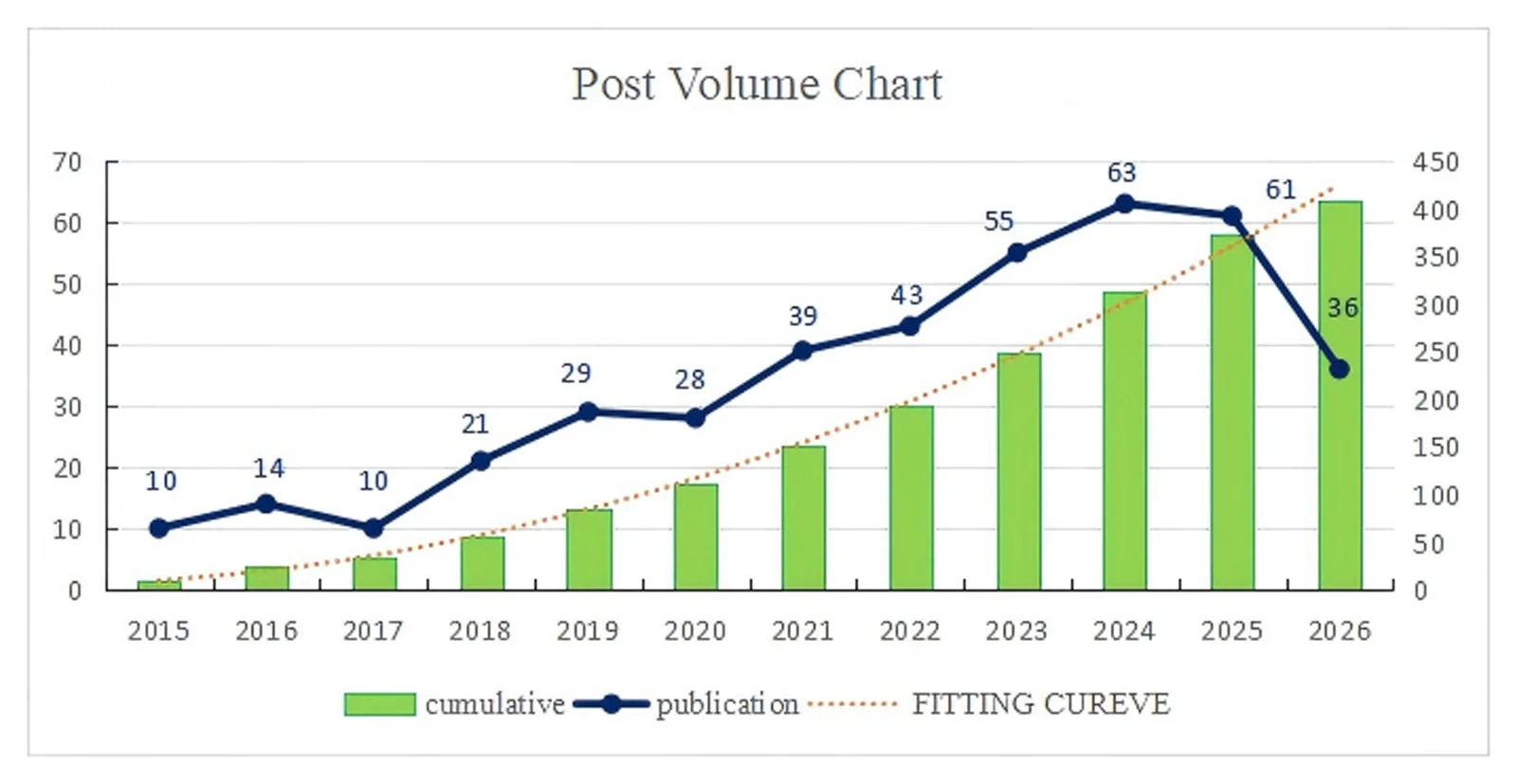
Annual publication trends of PNE studies. PNE = pain neuroscience education.

### 3.2. Analysis of national collaboration networks

The heatmap of publication output shows that the United States (US), Spain, and Belgium present highlighted red agglomeration zones, representing the 3 countries with the highest concentration of global PNE research output. Countries such as the United Kingdom and Australia surround the core zones with medium-yellow and orange rings, forming second-tier research forces. Asian, African, and Latin American countries, such as China, South Korea, and India, only form scattered light-blue low-heat spots with relatively limited overall publication volume, as shown in Figure 3. The global map of national research output uses blue gradients to characterize the total publication volume of each country: dark blue corresponds to nations with the largest publication volume, light blue represents low-output countries, and gray indicates regions with no relevant publications. The hierarchical distribution pattern on this map is consistent with the heatmap shown in Figure 4. The national geographical collaboration network reveals that multiple European countries form tight clusters, with dense links between Spain, Belgium, and other nations and frequent intra-regional academic exchanges. Developed countries in Europe and the US have established dense and stable transnational collaborations and occupy central hub positions in the global cooperation network, as illustrated in Figure 5.

**Figure 3.**
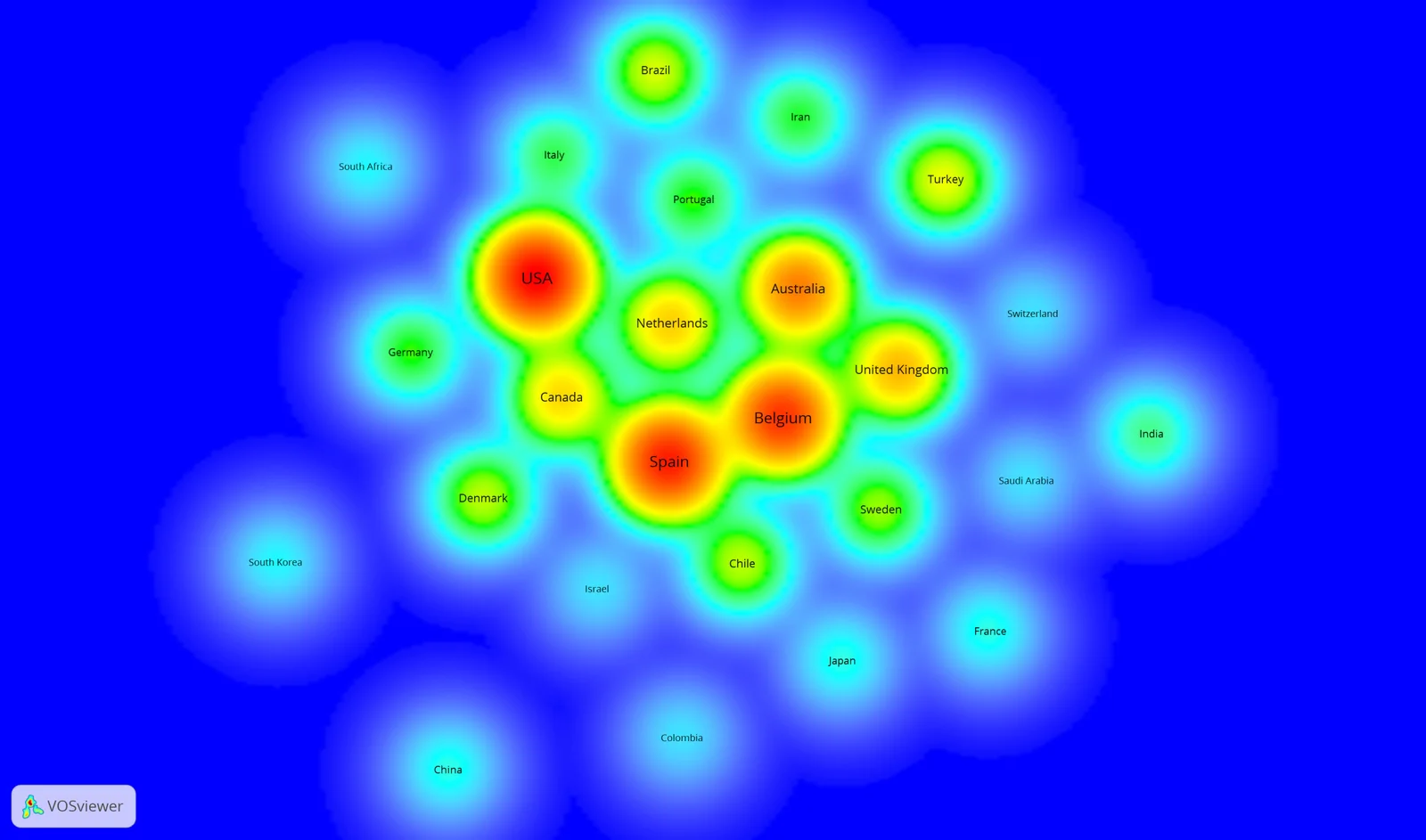
National output density heatmap of global PNE research. PNE = pain neuroscience education.

**Figure 4.**
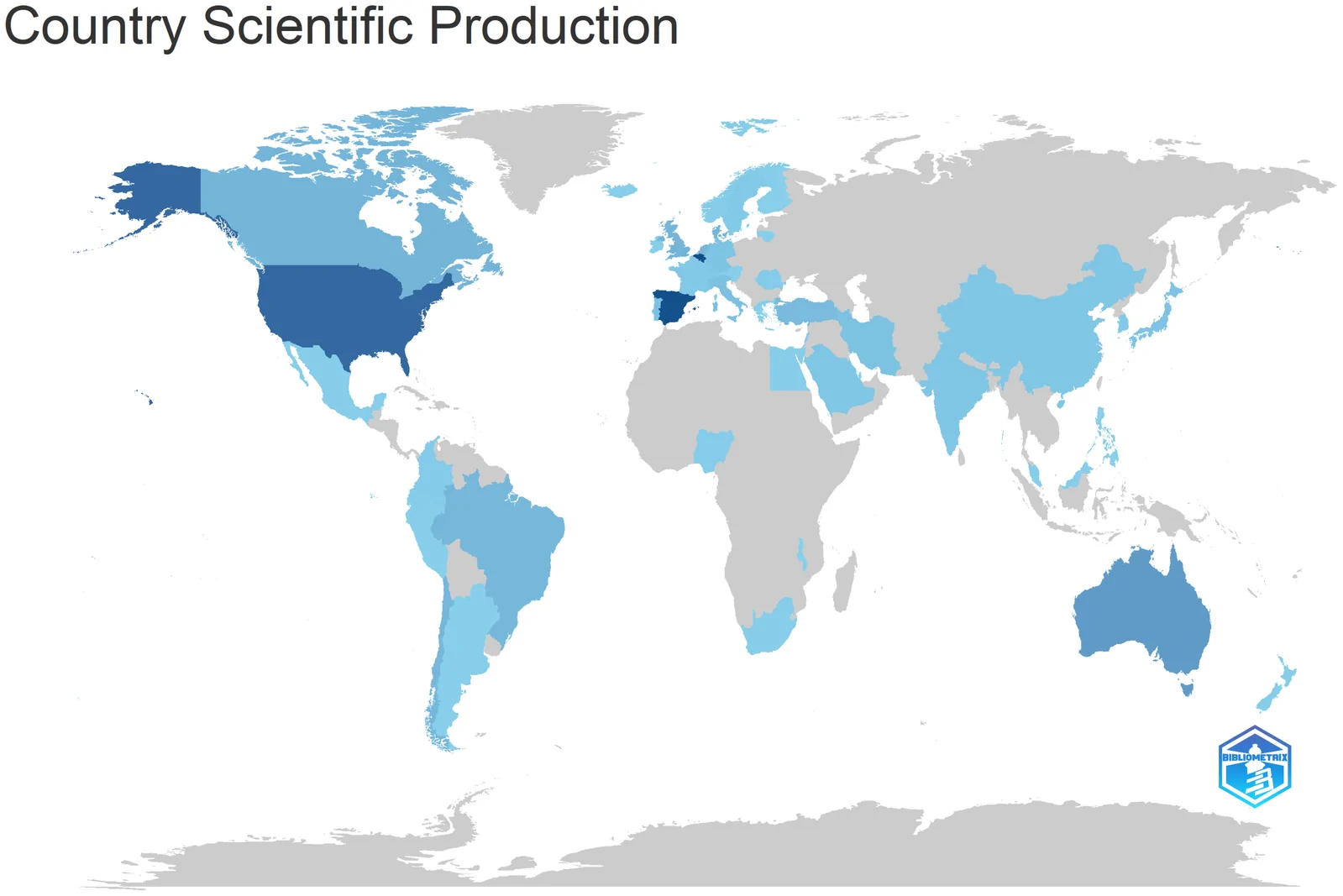
World map of national scientific production for PNE research. PNE = pain neuroscience education.

**Figure 5.**
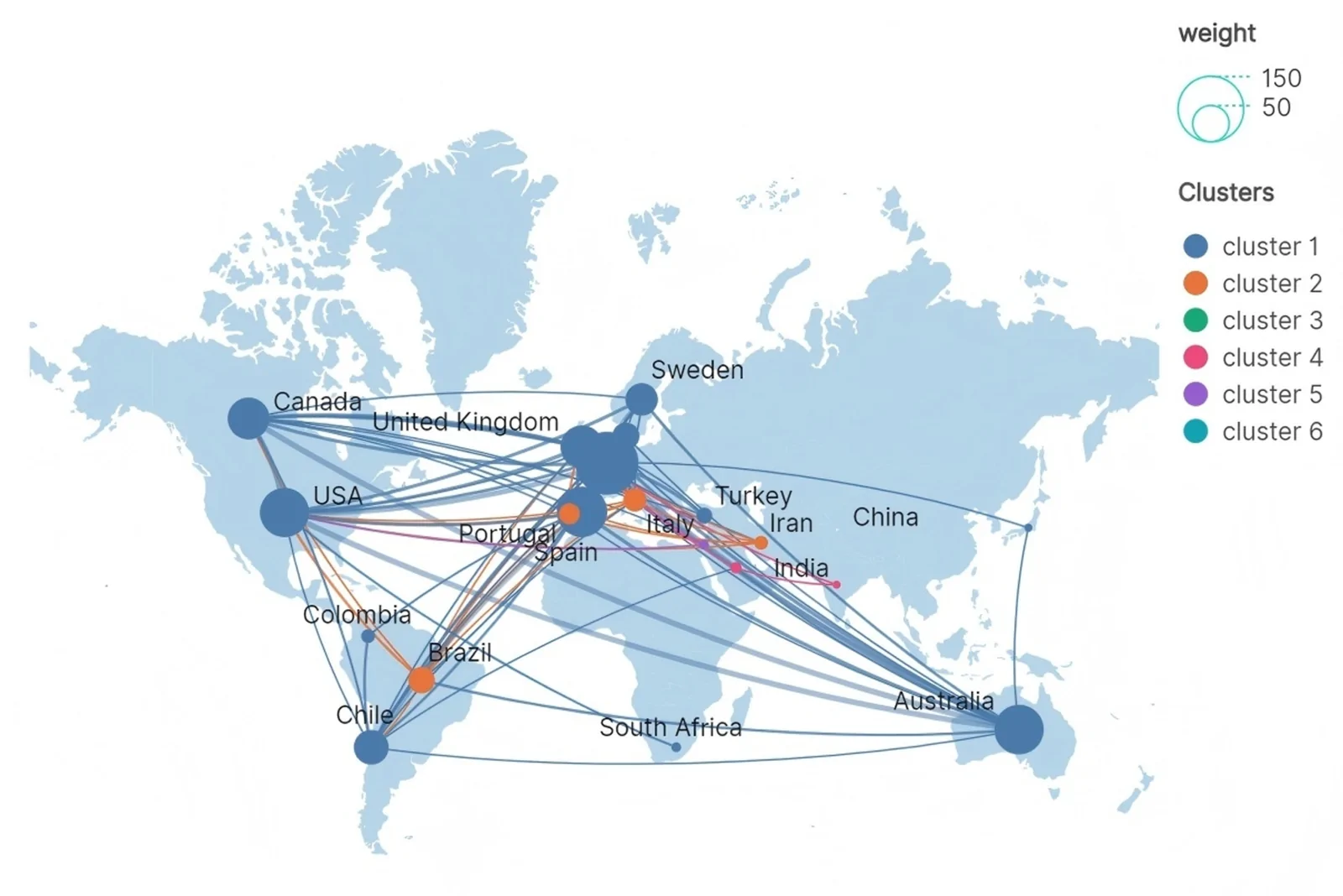
International country collaboration network.

### 3.3. Analysis of the author collaboration network

This network comprises 329 author nodes and 710 collaborative links, with a network density of 0.0132. In Figure 6, node size represents author output, and links between nodes indicate collaborative publication relationships; densely connected regions form several subnetworks representing different key research teams. Based on the core author calculation formula of Price Law,^[20]^ the threshold for high-output authors was calculated as 4.854; therefore, authors with ≥ 5 publications were classified as high-output authors. The results identified 23 high-output authors with a total of 267 publications. This proportion exceeds the theoretical 50% value predicted by Price Law, indicating that research output in the field of PNE is concentrated among a small number of prolific authors, and the leading role of this group is quite evident. The distribution of the top 10 high-output authors by publication volume is presented in Figure 7: Nijs J ranks first with 42 publications, followed by Meeus M (36 publications) and Louw A (28 publications), who form the second output echelon. These scholars jointly constitute the core research force of this field. An author co-occurrence clustering map was further constructed (Fig. 8). The network was automatically divided into 6 clusters distinguished by different colors. Close collaborations exist within each cluster, while links across teams and institutions are relatively sparse. This reveals stable internal cooperation within individual research groups, yet academic communication and collaboration between different teams need to be further strengthened.

**Figure 6.**
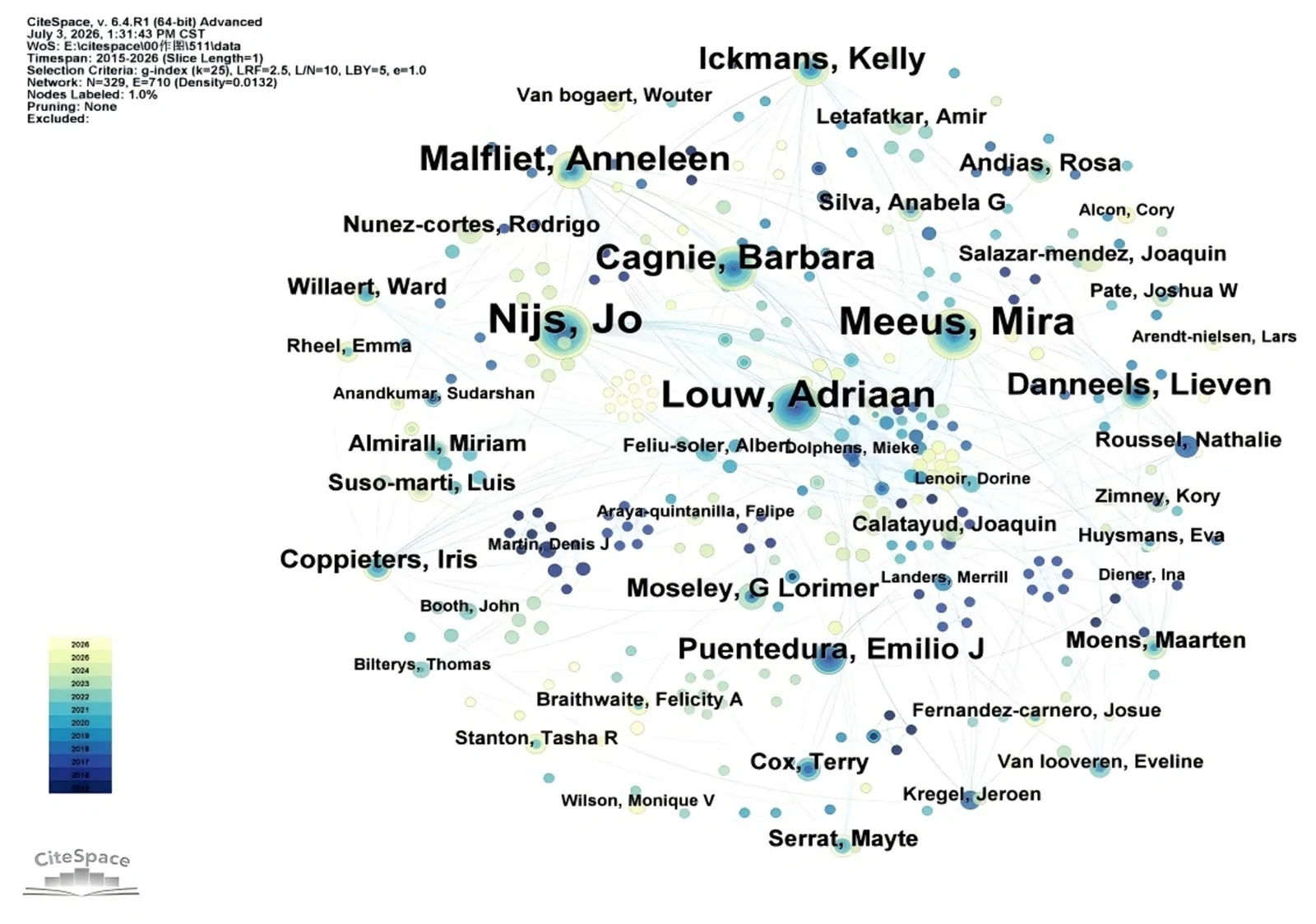
Author co-occurrence network generated by CiteSpace.

**Figure 7.**
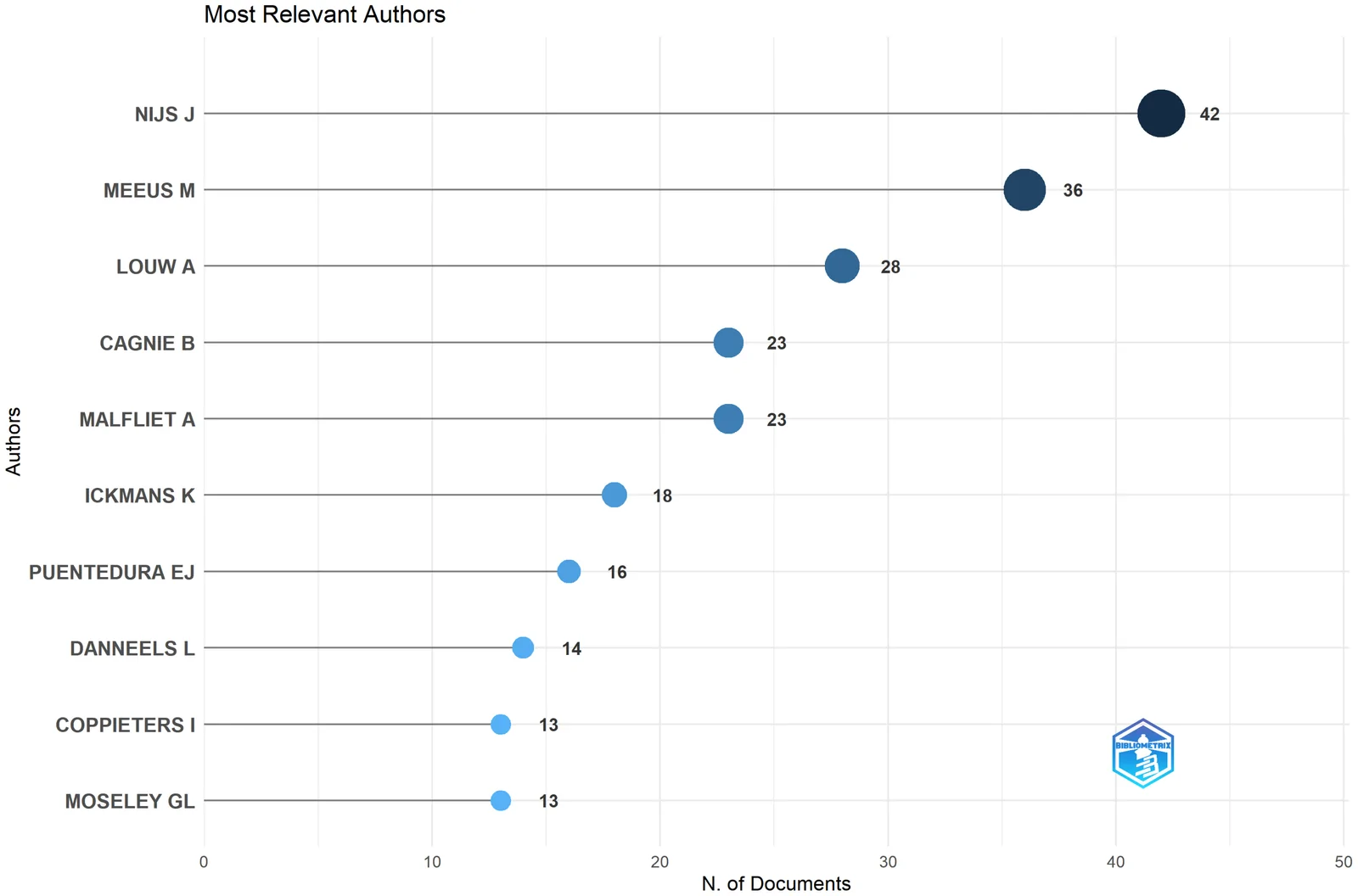
Horizontal bar chart of the top 10 productive authors.

**Figure 8.**
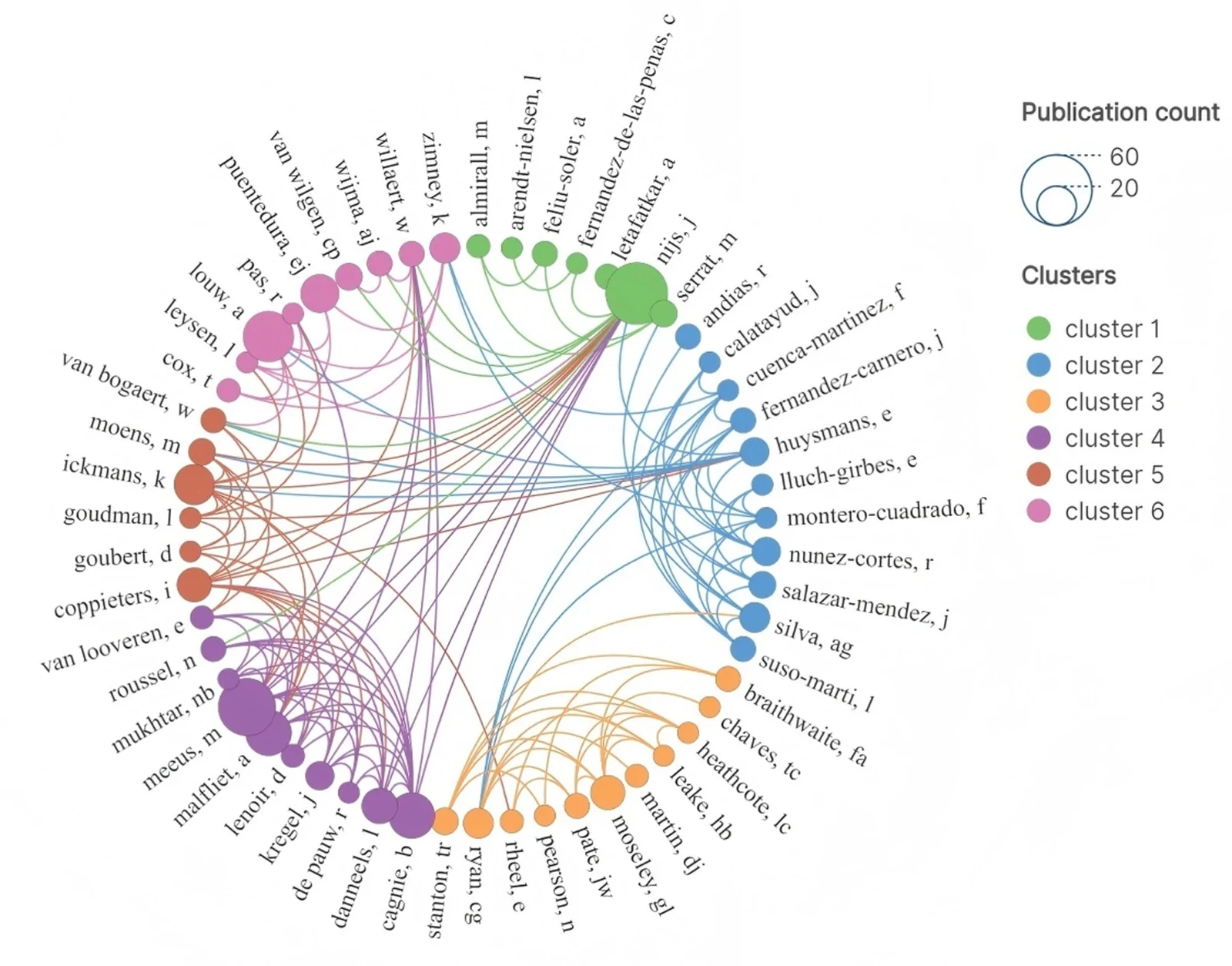
Author clustering network.

### 3.4. Keyword analysis

#### 3.4.1. Keyword co-occurrence analysis

Keyword co-occurrence analysis can visually reveal the distribution of hotspots and intellectual structure within a specific research field by counting the frequency of associated keyword appearances.^[21]^ For simplicity, synonyms and near-synonyms were merged; for example, “neuroscience pain education” and “neuroscience education” were combined and represented by the node “pain neuroscience education.” Spelling differences between British and American English were standardized to ensure node consistency. Keywords with a frequency of <2 were excluded. After running the analysis, the results yielded N = 342 nodes, E = 786 edges, and a network density of D = 0.0135, as shown in Figure 9. The top 5 keywords by frequency are “pain neuroscience education,” “low back pain,” “chronic pain,” “management,” and “chronic musculoskeletal pain”; the top 5 keywords by centrality are “anxiety,” “central sensitization inventory,” “physical therapy,” “cognitive behavioral therapy,” “beliefs” as shown in Tables 1 and 2. The VOSviewer network map uses a standard color gradient to indicate node aggregation density. The yellow area in the center of the map represents the highest-density region, where the above high-frequency keywords are concentrated; from the center outward, the color gradient shifts to green and then light blue, corresponding to low-density regions with low-frequency peripheral topics. This visualization allows intuitive differentiation between core research keywords and secondary branches in the field (Fig. 10).The keyword word cloud intuitively demonstrates the distribution of research topics in this field. As shown in Figure 11, pain neuroscience education, as the core keyword, appears with the highest frequency and a significantly larger font size than other terms. Chronic pain, chronic musculoskeletal pain, and low back pain constitute the second tier of high-frequency themes, forming the core clinical scenario of PNE research. Terms such as management, central sensitization, reliability, kinesiophobia, and exercise also occur frequently, reflecting that intervention management, mechanism interpretation, tool validation, and combined intervention are important research directions in this field.

**Table 1:** Top 5 keywords by frequency in chronic pain research on PNE.

Rank	Keyword	Frequency
1	pain neuroscience education	242
2	low back pain	190
3	chronic pain	143
4	management	89
5	musculoskeletal pain	85

PNE = pain neuroscience education.

**Table 2:** Top 5 keywords ranked by centrality in chronic pain research within the field of PNE.

Rank	Keyword	Mediation index
1	anxiety	0.26
2	central sensitization inventory	0.24
3	physical therapy	0.21
4	cognitive behavioral therapy	0.17
5	beliefs	0.15

PNE = pain neuroscience education.

**Figure 9.**
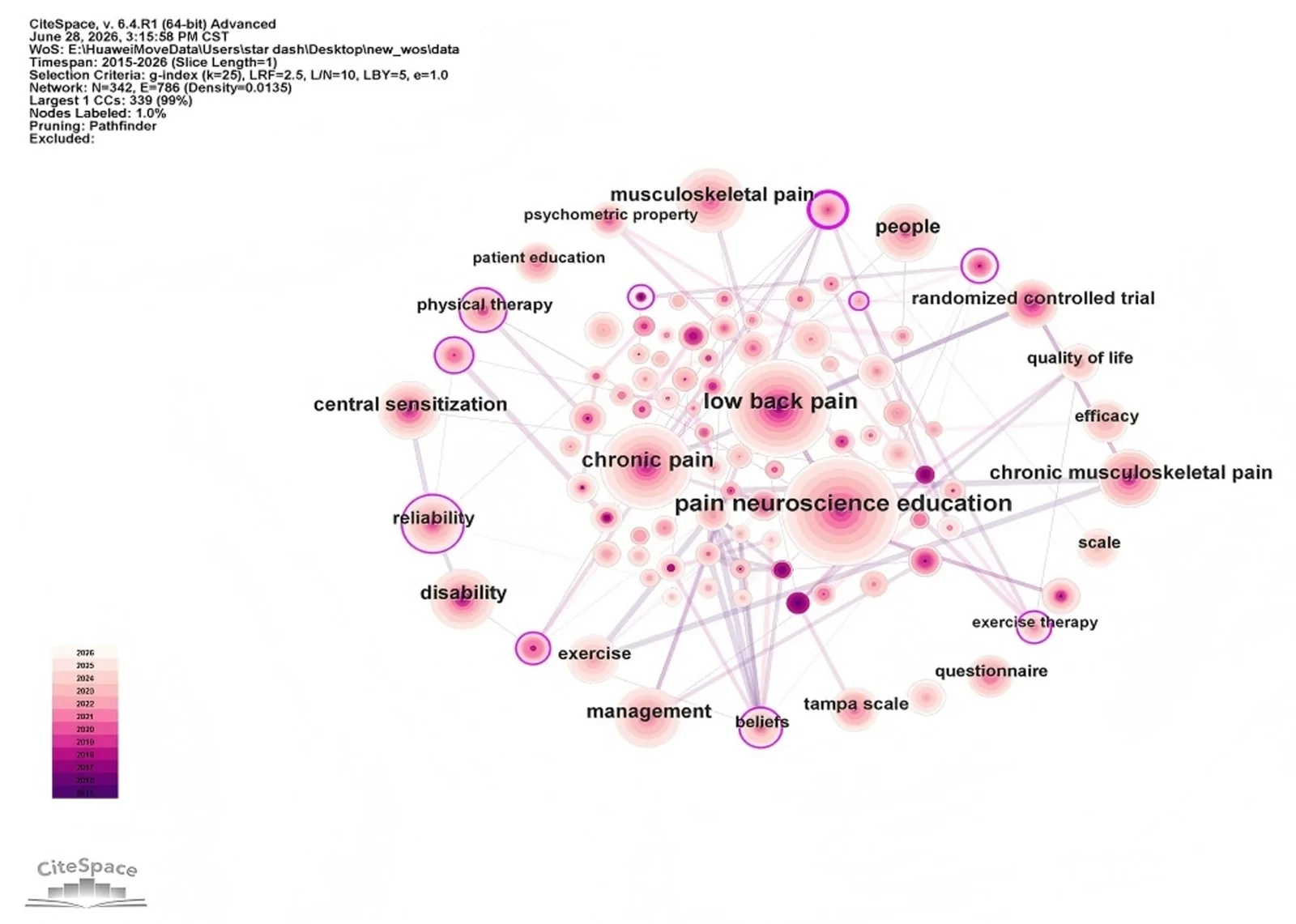
Keyword co-occurrence network via CiteSpace.

**Figure 10.**
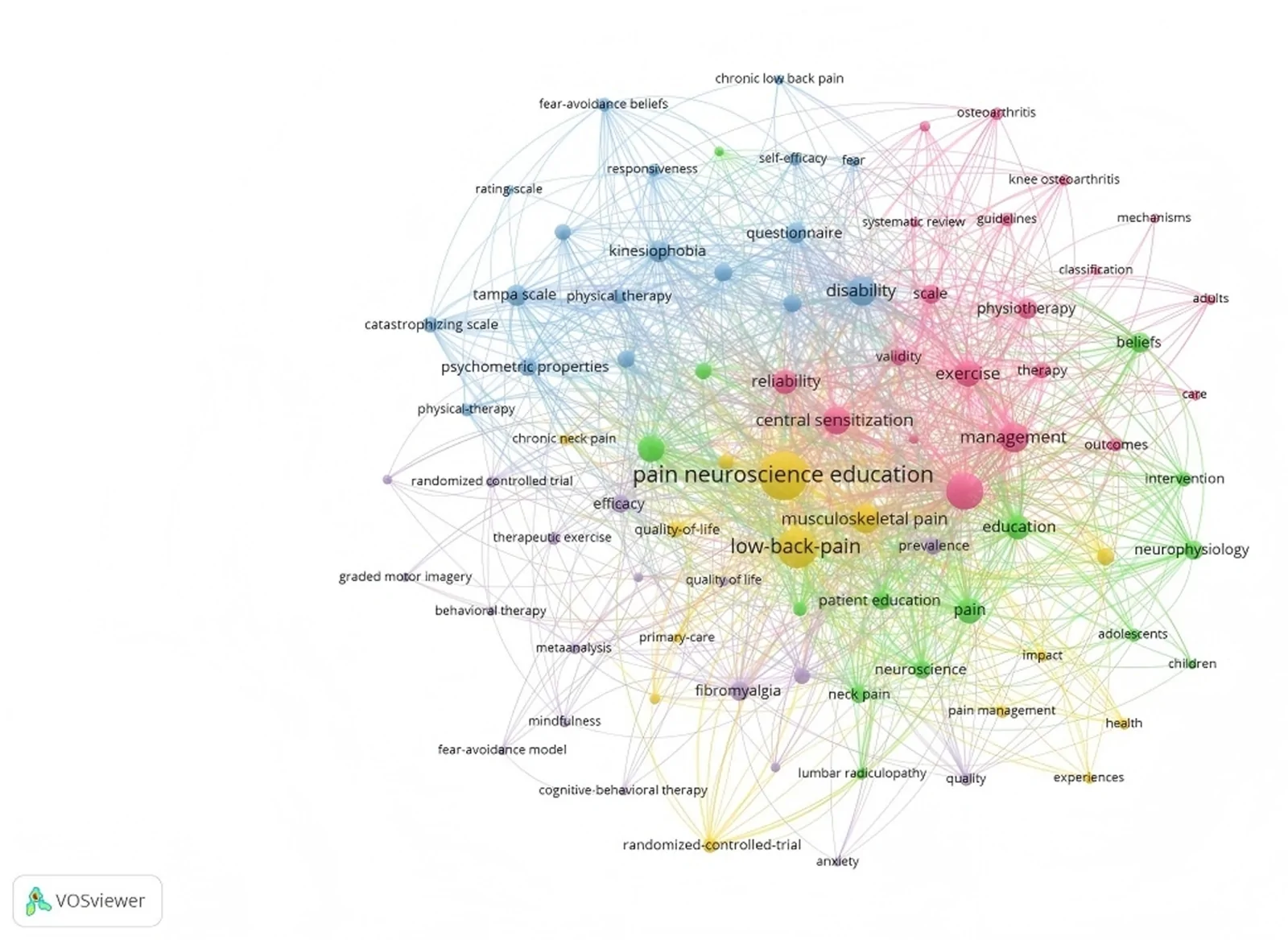
Keyword density clustering map produced by VOSviewer.

**Figure 11.**
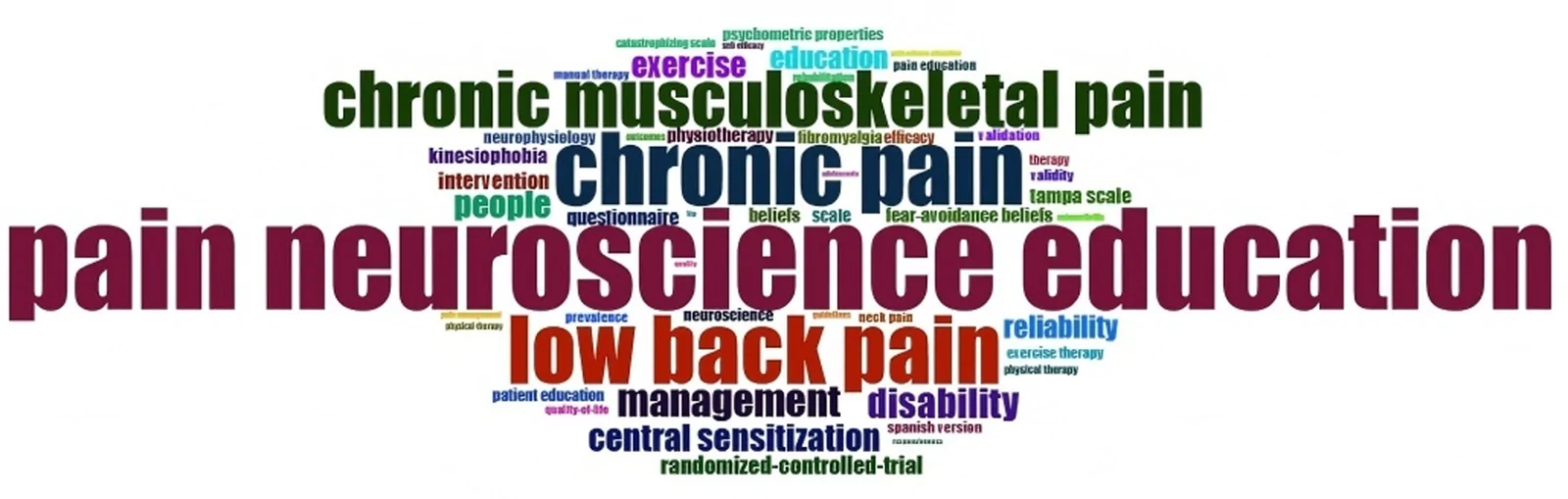
High-frequency keyword word cloud.

#### 3.4.2. Keyword cluster analysis

Cluster analysis of the keywords was performed using the log-likelihood ratio algorithm. The top 10 clusters were selected and visualized, as shown in Figure 12 and Table 3. The cluster modularity value *Q* = 0.6982 (> 0.3) indicates that the network possesses a significant clustering structure. The average silhouette value *S* = 0.8788 (> 0.7) reflects strong internal homogeneity within each cluster, indicating high reliability of the clustering results. Overall, the keyword clustering yielded good results with a clear and stable structure, effectively reflecting the distribution of research themes in this field. Cluster #0, the largest cluster in the network, focuses on neural mechanisms and the biopsychosocial model of pain. In terms of treatment dimensions, #2 dry needling, #3 exercise therapy, and #7 cognitive behavioral therapy represent physical, exercise, and psychological intervention modalities, respectively, while #9 interoceptive exposure is associated with behavioral addiction and behavioral health. At the research subject level, #1 chronic pain and #8 low back pain form the fundamental research symptoms in this field. Additionally, #5 feasibility study and #6 pain management reflect a growing trend toward clinical translation and implementation science in PNE research.

**Table 3:** Keyword cluster analysis of PNE in chronic pain research.

Cluster ID	Cluster name	Coefficient of similarity	Primary keywords
#0	pain neuroscience education	0.886	neurobiological mechanisms; psychosocial factors; biopsychosocial model; narrative medicine; nonspecific chronic
#1	chronic pain	0.917	musculoskeletal pain; psychological distress; chronic low back pain; home exercises; pain intensity
#2	dry needling	0.875	trigger point; pain-related fear; central sensitization; neuropathic pain; whole-body cryostimulation
#3	exercise therapy	0.896	low back pain; body weights; chronic spinal pain; mediation analysis; manual therapy
#4	physical therapy	0.881	cognitive behavioral therapy; manual therapy; musculoskeletal pain; language discordance; straight leg raise
#5	feasibility study	0.846	education; pain beliefs; chronic spinal pain; multiple mediators; treatment protocol
#6	pain management	0.821	patient education; pain intensity; university students; health education; spinal manipulative therapy
#7	cognitive behavioral therapy	0.766	virtual reality; low back pain; graded exposure; occupational health; cognitive behavioral therapy
#8	low back pain	0.849	exercise therapy; physiotherapy; management; people; randomized controlled trial
#9	interoceptive exposure	0.91	interoceptive exposure; behavioral health; occupational health; graded exposure; behavioral addiction

PNE = pain neuroscience education.

**Figure 12.**
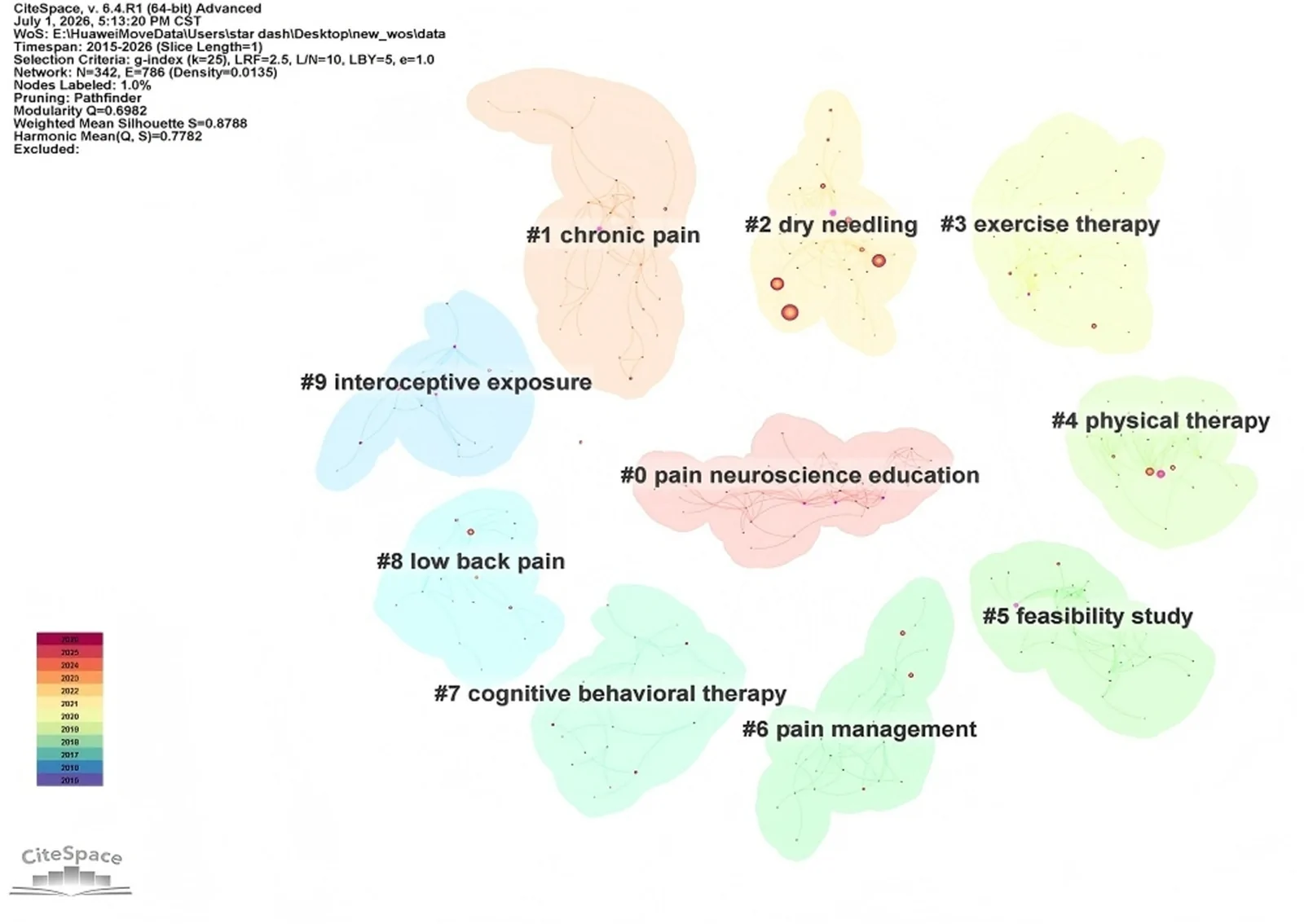
Keyword clustering map.

#### 3.4.3. Keyword burst analysis

Based on the results of keyword co-occurrence and clustering analysis, further keyword burst analysis was performed. By identifying keywords with sharply increased frequency within a specific period, this approach revealed the evolutionary dynamics and research trends of hot topics in the field. A total of 25 emerging keywords were identified. Keywords with high burst strength included chronic whiplash, randomized controlled trials, chronic fatigue syndrome, physiotherapy, and physiology education, as shown in Figure 13.

**Figure 13.**
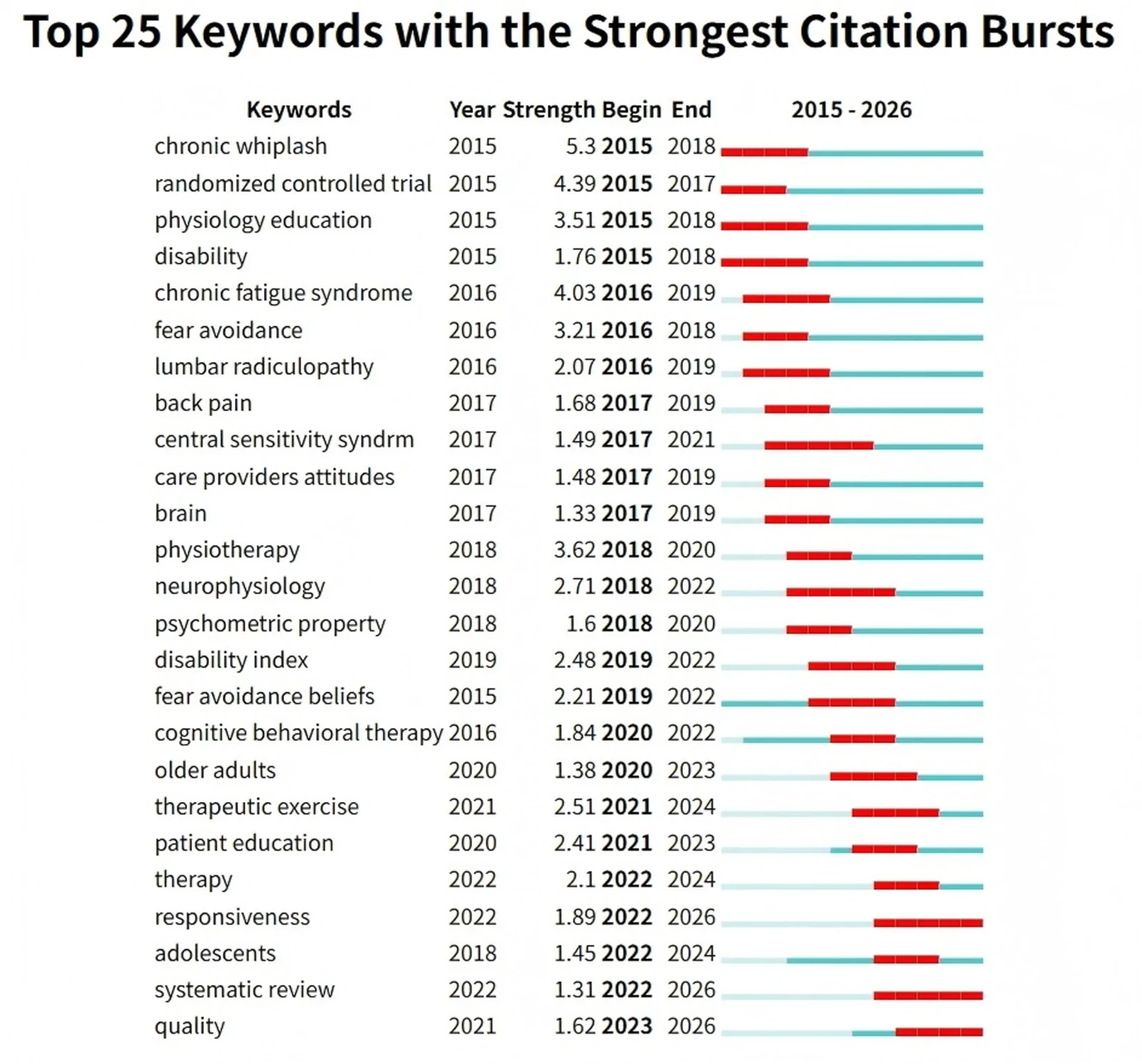
Top 25 keywords with citation bursts.

### 3.5. Citation frequency analysis

Highly co-cited references reflect academic consensus within the field, serve as key drivers of research evolution, and clarify the guiding value of relevant findings for subsequent research. The top 10 most frequently co-cited papers, ranked by citation frequency, are shown in Table 4. Core literature in this field was primarily published between 2015 and 2022, covering studies such as systematic reviews and randomized controlled trials. The most frequently cited paper is a mixed-methods review by Watson et al, published in The Journal of Pain. This paper provides a comprehensive analysis of PNE for the treatment of chronic musculoskeletal pain and objectively evaluates the efficacy of interventions, making it a highly recognized classic work in the field. The 3-field plot illustrates the knowledge flow among highly cited references, authors, and research topics (Fig. 14). The highly cited references on the left are mostly classic empirical studies and reviews in the field of PNE. Among the authors in the middle column, scholars including Louw A, Nijs J, Moseley GL, and Meeus M show strong links with multiple highly cited references. The keywords on the right indicate that the research outputs of the aforementioned scholars mainly converge on topics such as low back pain, central sensitization, PNE, and chronic musculoskeletal pain, which collectively constitute the knowledge backbone of this field.

**Table 4:** Analysis of highly cited papers.

Rank	Publication	Citation count	Journal	Year
1	Pain Neuroscience Education for Adults With Chronic Musculoskeletal Pain: A Mixed-Methods Systematic Review and Meta-Analysis	87	The Journal of Pain	2019
2	Short-term impact of combining pain neuroscience education with exercise for chronic musculoskeletal pain: a systematic review and meta-analysis	63	Pain	2022
3	A systematic review and meta-analysis of pain neuroscience education for chronic low back pain: Short-and long-term outcomes of pain and disability	59	European Journal of Pain	2019
4	The efficacy of pain neuroscience education on musculoskeletal pain: A systematic review of the literature	50	Physiotherapy Theory and Practice	2016
5	Effect of Pain Neuroscience Education Combined With Cognition-Targeted Motor Control Training on Chronic Spinal Pain: A Randomized Clinical Trial	44	JAMA Neurology	2018
6	Fifteen Years of Explaining Pain: The Past, Present, and Future	37	The Journal of Pain	2015
7	Pain Neurophysiology Education and Therapeutic Exercise for Patients With Chronic Low Back Pain: A Single-Blind Randomized Controlled Trial	36	Archives of Physical Medicine and Rehabilitation	2018
8	Pain Neuroscience Education and Physical Therapeutic Exercise for Patients with Chronic Spinal Pain in Spanish Physiotherapy Primary Care: A Pragmatic Randomized Controlled Trial	30	Journal of Clinical Medicine	2020
9	Blended-Learning Pain Neuroscience Education for People With Chronic Spinal Pain: Randomized Controlled Multicenter Trial	28	Physical Therapy	2018
10	How Much Is Needed? Comparison of the Effectiveness of Different Pain Education Dosages in Patients with Fibromyalgia	27	Pain Medicine	2020

**Figure 14.**
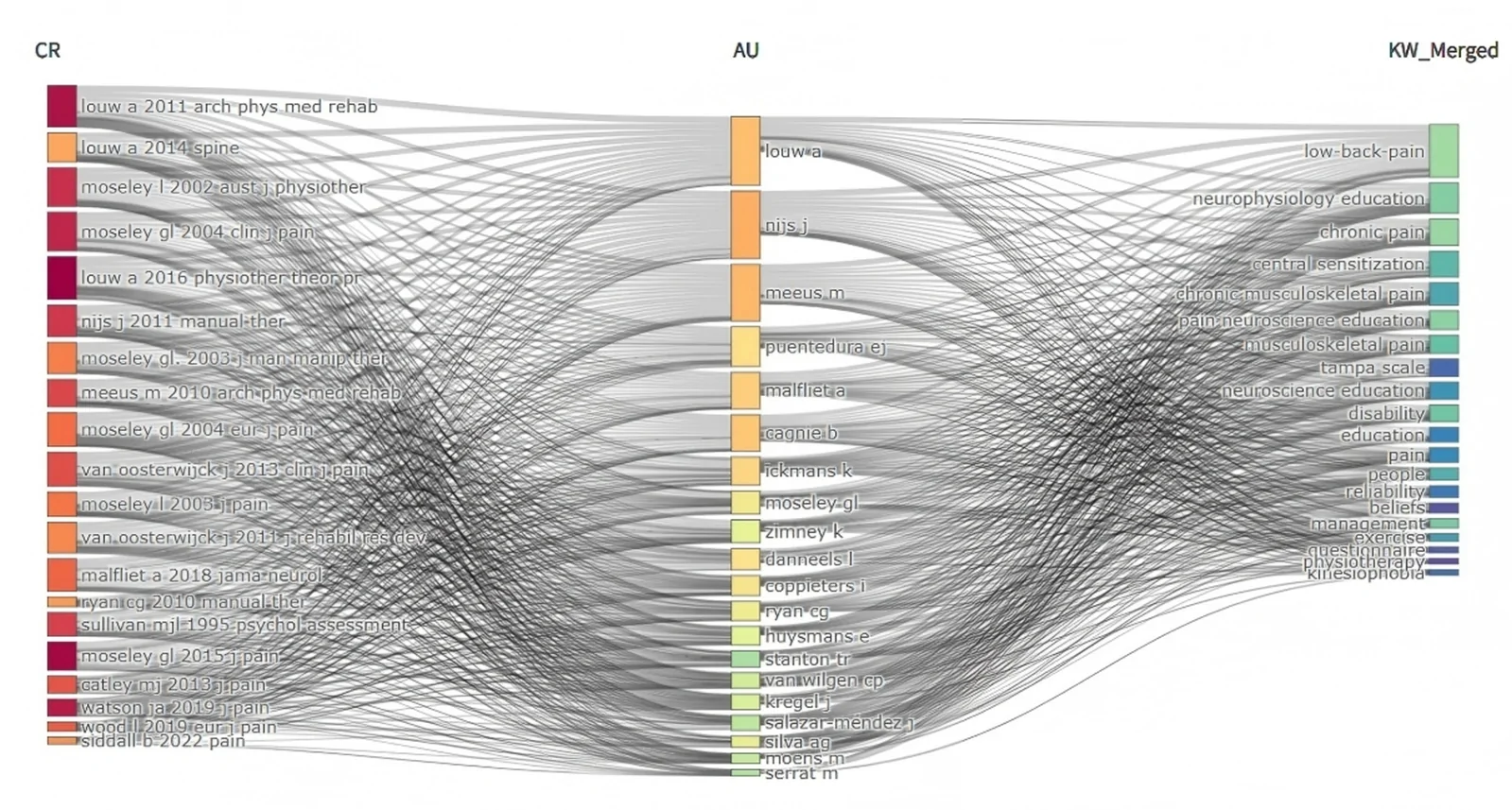
Three-field plot. AU = authors, CR = cited references, KW = keywords.

## 4. Discussion

### 4.1. Current state of research

The annual output of publications in the field of PNE has grown rapidly since 2018, peaking in 2024, reflecting increased attention from researchers worldwide and establishing the field as a research hotspot. This growth trend aligns with the recent shift in pain management from the traditional biomedical model to the biopsychosocial model, as well as the trend in academic research toward in-depth exploration of the mechanisms underlying human pain experiences.

Analysis of national collaboration networks indicates that PNE research output is primarily led by Western developed countries, which have formed relatively stable collaborative relationships with one another. In contrast, countries in Asia, Latin America, and other low- and middle-income regions show generally low publication productivity and low node betweenness centrality, occupying marginal positions in the global collaboration network with considerable room for improvement in international participation and academic influence. These disparities may be attributable to regional differences in the acceptance of the biopsychosocial pain model within healthcare systems, uneven popularization of modern pain neuroscience concepts among frontline clinicians, and the relative scarcity of localized PNE implementation protocols and educational resources in non-Western contexts.^[22,23]^

The US has the highest PNE research output globally, a phenomenon that can be attributed to 2 key factors. First, driven by substantial public health demands, the North American opioid crisis has imposed severe health and economic burdens, prompting clinical sectors to seek non-pharmacological analgesic alternatives.^[24,25]^ Second, policy drivers have accelerated relevant research: the 2016 Centers for Disease Control and Prevention Guideline for Prescribing Opioids for Chronic Noncancer Pain and the subsequent National Pain Strategy have prioritized non-pharmacological interventions as first-line approaches for chronic pain management,^[26]^ providing clear policy guidance for PNE-related studies. Future research should strengthen cross-regional academic collaboration, promote bidirectional knowledge translation, and develop culturally adapted and clinically applicable PNE protocols to improve the generalizability and extrapolation of research findings.

Analysis of author collaboration networks indicates that several research teams led by highly productive authors have formed within the field and collaborate closely, consistent with the centralization of research activity described by Price Law. These teams play a leading role in promoting the standardization of PNE intervention protocols and in exploring the mechanisms of efficacy. Specifically, the Jo Nijs team with the highest publication output conducts comprehensive research covering the full research pipeline from central sensitization mechanisms to clinical implementation of PNE, ensuring sustained research focus and high productivity. This team first proposed clinical identification criteria for central sensitization, laying a foundation for standardized research in this field,^[19]^ and further conducted high-quality mechanism-verifying randomized controlled trials, providing high-level evidence for the efficacy of PNE.^[27]^ However, the relatively low network density also suggests that cross-institutional and cross-regional collaboration among different teams remains insufficient. Future research should prioritize inter-team collaboration to foster broader academic exchange and cooperation.

Due to factors including sub-disciplinary research output and the duration of literature publication, the highest citation count observed among the included papers was 87, with overall citation counts remaining relatively low. Temporal analysis of highly cited literature reveals that early studies mainly focused on verifying the efficacy of single PNE interventions, whereas recent studies have increasingly emphasized combined intervention effects, dosage optimization, and implementation models. These highly cited publications constitute the core knowledge base of the field and reflect the evolving research priorities, facilitating the standardization and precision of clinical PNE application.

### 4.2. Research Hotspots

#### 4.2.1. Application of PNE in Chronic Musculoskeletal Pain

Keyword analysis reveals that “low back pain” and “chronic musculoskeletal pain” are high-frequency and clustered keywords, indicating that current PNE research and practice are primarily focused on patients with chronic musculoskeletal pain.This aligns with the high global prevalence and disability rate of such pain, as well as its close association with complex biopsychosocial mechanisms (including significant central sensitization and pain catastrophizing) that underlie its onset and maintenance.^[28]^ Accordingly, PNE is well-suited to tackle the complexity of musculoskeletal pain by interpreting the neural plasticity mechanisms of pain. In terms of evidence accumulation, early efficacy verification studies of PNE mainly targeted chronic low back pain, forming a sufficient evidence base and prompting further in‑depth investigation in this field.^[29]^ Although numerous studies have focused on musculoskeletal pain, the application of PNE to other types of chronic pain remains limited. For instance, several preliminary exploratory studies have investigated PNE in neuropathic pain and cancer-related pain,^[30–32]^ but high-quality clinical evidence and systematic reviews are still insufficient. This gap may be attributed to the mechanistic heterogeneity among different pain types, which challenges the generalizable interpretive framework of PNE. Specifically, anticipated disease progression and persistent tissue damage linked to cancer pain may reduce the applicability of the core PNE tenet that “pain does not equal tissue damage.” Future studies are urgently required to explore the applicable boundaries of PNE for pains of different etiologies and develop differentiated educational content and delivery strategies for distinct pain types. In clinical practice, healthcare professionals should integrate PNE into the whole-course management of chronic pain patients. Incorporating PNE during patient admission education can help patients establish rational pain perceptions and ensure continuous educational support in routine pain management.

#### 4.2.2. Optimizing intervention models from single-dimensional education to comprehensive programs

Keyword cluster results show that intervention modalities, including exercise therapy, cognitive behavioral therapy, and dry needling, form independent clusters, indicating that the research focus has shifted from the single efficacy verification of standalone PNE to the optimization of multimodal combined intervention programs, which reflects the upgrading of chronic pain management concepts. Chronic pain is a complex disorder influenced by biological, psychological, and social factors, and a single cognitive-oriented intervention fails to fully cover its multidirectional pathogenic mechanisms. Previous studies have confirmed that standalone PNE yields limited short-term improvements in pain intensity. In contrast, when PNE is combined with exercise therapy, peripheral proprioceptive input generated by exercise can further consolidate central neuroplastic changes and produce synergistic effects.^[33]^ The combination of PNE and cognitive behavioral therapy can specifically ameliorate pain catastrophizing, anxiety, and other psychological comorbidities, thereby improving long-term intervention efficacy.^[34]^ Early studies predominantly adopted a basic combination of PNE and exercise therapy. In recent years, multimodal comprehensive intervention schemes integrating PNE with exercise therapy, cognitive behavioral therapy, and mindfulness have gradually emerged.^[35,36]^ Intervention scenarios have extended from in-hospital settings to communities, homes, and online platforms,^[30,37,38]^ which conforms to the concept of whole-process chronic pain management.

Nevertheless, several research gaps remain, particularly the unclear dose–response relationship of PNE interventions. The optimal duration, frequency, and presentation modes of PNE, as well as the optimal proportion of each component in combined interventions, have received insufficient research attention.^[11]^ Furthermore, current study designs are mostly unable to clarify the effective mechanisms underlying combined protocols: whether the therapeutic effects are driven by PNE-induced cognitive modifications, physiological benefits of exercise, or genuine synergistic interactions between different interventions. Future research should adopt factorial designs, mediation effect analysis, and other analytical methods to identify the key effective components of multimodal intervention programs, so as to realize precise and individualized PNE intervention strategies.

#### 4.2.3. Assessment tools and methodological validation

Keyword betweenness centrality analysis shows that keywords such as “reliability” and the “central sensitization inventory” rank high, demonstrating that methodological research on assessment tools acts as a critical node connecting diverse research directions and lays an essential foundation for the standardized development of this field. Early studies mainly adopted pain intensity and functional disability as primary outcome indicators. The absence of standardized instruments for evaluating changes in pain cognition following PNE interventions has led to high heterogeneity and poor comparability of evidence across different studies. Accordingly, researchers have begun to conduct cross-cultural adaptation, reliability, and validity verification for tools, including the Central Sensitization Inventory, Pain Neuroscience Knowledge Questionnaire, and Fear-Avoidance Beliefs Questionnaire, providing methodological support for standardized research in this domain. Meanwhile, outcome indicators have gradually expanded from single pain intensity measurements to multidimensional assessments covering pain cognition, kinesiophobia, psychological status, and quality of life. This transition not only broadens the scope of evaluation indicators but also reflects an updated academic consensus on judging PNE intervention efficacy. Rather than being judged merely by reductions in pain intensity scores, therapeutic outcomes are now assessed according to substantial improvements in patients’ pain coping patterns, consistent with the holistic evaluation philosophy of the biopsychosocial model.^[39,40]^

### 4.3. Research trends

Keyword burst analysis shows that the keyword “systematic review” has exhibited high burst strength in recent years. This finding reveals that numerous original empirical studies on PNE for chronic pain have accumulated, and the whole field has entered the stage of evidence-based synthesis. Researchers have integrated scattered clinical trials via systematic reviews and meta-analyses, unified the implementation specifications of interventions and research reporting standards, and generated high-level synthesized evidence. These efforts provide evidence-based support for incorporating PNE into clinical guidelines and establishing standardized pain diagnosis and treatment pathways, thereby facilitating the transformation of PNE from a research hotspot to a routine clinical practice. This developmental characteristic is also consistent with the large number of umbrella reviews and meta-analyses recently published in *The Journal of Pain* and *Annals of Physical and Rehabilitation Medicine*.^[41,42]^

In addition, the emergence of “therapeutic exercise” and “patient education” further supports the trend toward multimodal combined interventions identified in the previous cluster analysis. The burst keywords “older adults” and “adolescents” indicate that age-stratified analyses of individualized interventions are required in future research. Elderly patients often present with special factors including multimorbidity, varying cognitive function, and modified social support systems, while adolescents are characterized by high neurodevelopmental plasticity and heavy academic and social adaptation pressure. Thus, differentiated PNE content, educational approaches, and delivery media should be adopted for these 2 populations. Meanwhile, most current reporting standards for PNE research have been developed for adults, and supplementary reporting criteria for interventions targeting older adults and adolescents remain inadequate.

## 5. Conclusions

In summary, PNE has emerged as a rapidly growing research hotspot with extensive international collaboration in the field of chronic pain management. Current PNE research adopts the biopsychosocial model as its framework. Multimodal interventions and the validation of assessment tools are attracting increasing attention, alongside improvements in methodological quality and evidence synthesis. Nevertheless, the geographical distribution of relevant studies remains unbalanced. Developed countries in Europe and North America dominate global research and have formed dense and stable transnational collaboration networks, while academic participation and international influence in Asia, Latin America, and low- and middle-income regions remain relatively limited. Future research should aim to strengthen cross-regional knowledge translation and cooperation, develop implementation strategies tailored to diverse cultural backgrounds and medical resource allocation, and enhance the transferability and global applicability of research findings, ultimately helping more patients with chronic pain relieve suffering and improve their quality of life.

This study has several limitations: only the WoSCC was retrieved, leading to incomplete literature coverage; literature data for 2026 were incomplete; bibliometric analysis was descriptive and could not assess intervention efficacy or causal relationships; parameter setting and node processing in CiteSpace, VOSviewer, and Bibliometrix carried a certain subjectivity; and the inherent limitations of these algorithms cannot be completely eliminated. Follow-up research can further optimize the analysis by expanding the retrieval databases and refining the search strategies.

## Acknowledgments

The authors sincerely thank all researchers whose publications were included in this bibliometric analysis. We are grateful to the reviewers for their valuable comments and suggestions to improve this manuscript.

## Author contributions

**Conceptualization:** Yixin Chen, Huamei Yu.

**Data curation:** Yixin Chen, Lixian Dang.

**Formal analysis:** Yixin Chen, Lixian Dang.

**Investigation:** Yixin Chen, Lingling Li, Chenyang Li.

**Methodology:** Yixin Chen, Lixian Dang.

**Project administration:** Huamei Yu.

**Software:** Yixin Chen, Lingling Li.

**Supervision:** Huamei Yu.

**Validation:** Huamei Yu, Chenyang Li.

**Visualization:** Yixin Chen.

**Writing – original draft:** Yixin Chen.

**Writing – review & editing:** Huamei Yu, Lingling Li, Chenyang Li.
